# Lobar-Pneumonia, an Acute Specific Fever

**Published:** 1899-04-22

**Authors:** J. O. Symes

**Affiliations:** Bacteriologist, Bristol Royal Infirmary


### Hospital Clinics and Medical Progress.

### LOBAR-PNEUMONIA, AN ACUTE SPECIFIC FEVER.

By J. O. Symes, M.D., D.P.H., Bacteriologist, Bristol Royal Infirmary.

Lobar-pneumonia is to be distinguished from other inflammatory conditions of the lung, such as broncho-catarrhal-, septic-, and hypostatic-pneumonia. Clinically this distinction is not easy, especially when a large area of the lung is involved, but much light may be thrown upon the case by a bacteriological examination. If on microscopical examination the sputum be found to contain pneumococci in large numbers, the case is probably one of true pneumonia, whilst if strepto- or staphylococci be in excess then the probability is that the disease is a broncho-pneumonia. Such examination of the sputum is, however, not entirely satisfactory, for the sputa may be contaminated during passage through the upper air passages and mouth. It is therefore better to directly examine the material of the hepatised lung. The skin over a consolidated portion of lung is cleansed by first washing with turpentine, and then with some efficient antiseptic solution, and the needle of a hypodermic or exploring syringe (previously sterilised by boiling) is then thrust into the lung, and a few drops of sanguineous fluid obtained. Examination of material so obtained will at once solve all doubts as to the nature of the disorder; that is, if it be granted that true lobar pneumonia is an inflammation of the lung due to the invasion of the pneumococcus. But the disease pneumonia is more than a pulmonary disorder. It is to be regarded as an acute specific fever. Clinically, pneumonia resembles the specific fevers in the mode of its onset, in the course taken by the fever, and in the nature of the complications. Direct infection from one person to another is rare, but epidemics of pneumonia are not uncommon. The almost constant presence of a distinctive microbe is perhaps the most striking proof of the specific nature of the disease, and the fact that the pneumococcus may be found in healthy sputum, and is sometimes associated with staphylo- and streptococci in cases of broncho-pneumonia in no way negatives this view.

The pyrexia of this fever may exist before there are any signs of lung implication, and such signs may never arise. Clinically it is not uncommon for symptoms of pneumonia to appear some hours or days before physical signs of lung consolidation can be demonstrated. The pneumococcus invasion may altogether spare the lung, and the disease manifest itself as a pleurisy, as a tonsillitis, or as a middle ear suppuration. More commonly, together with pulmonary hepatisation, the commonest symptom of this infective process, there are other symptoms—pericarditis, otitis, meningitis, ulcerative endocarditis, ulcerative colitis, or empyema—a long list, which forcibly indicates the widespread nature of the infection. The following cases which I have recently examined serve to illustrate this point:—

Case 1. The immediate cause of death was pneumonia, but in addition there was suppurative peritonitis and otitis media, and in all the lesions pneumococci were present.

Case 2. Pneumonia, with double otitis media. Cultures taken after death from pus from the ears, from the consolidated lung, and the spleen all gave rise to pneumococci.

Case 3. Pneumonia, with suppurative pericarditis, both of pneumococcal origin. Pneumococcus also isolated from spleen.

Case 4. Pneumonia, general meningitis, and purulent otitis media. The pneumococcus was present in all the exudations.

The suppurative lesions excited by the pneumococcus do not necessarily arise during the acute stage of pneumonia, but may occur when convalescence is well established. It is not necessary to explain their occurrence as being due to septic emboli, for in the more severe cases certainly, and probably in all cases of pneumonia, the specific organism is to be found in the blood. Their presence may be demonstrated by drawing off, with strict antiseptic precautions, a small quantity of blood from a vein of the fore-arm, and examining it either in films or by means of cultures. The presence, too, of the pneumococcus in the spleen when examined after death is a certain proof of its wide distribution by means of the general circulation. Apart from the bacteriological side of the question, there is a striking piece of chemical evidence pointing to the specific nature of the pneumococcus infection. In many of the acute specific fevers there is during the period of pyrexia a striking diminution of the chlorides in the urine. This is very marked in lobar-pneumonia, in which disease the chlorides may be entirely absent during the stage of fever, to return only some days after the crisis. This absence or diminution of the chlorides of the urine is not observed in broncho-pneumonia, in pleurisy, or in empyema, and may be regarded as a diagnostic sign of the specific lung infection. The crisis in pneumonia may be due to one of two causes. Either the life activity of pneumococcus has ceased, or a quantity of antitoxin has been formed sufficient to neutralise the toxin already in the circulation. In either case the resemblance to the course of the specific fevers is obvious. We have not at present sufficient evidence to decide as to the value of the antitoxin treatment of pneumonia. The serum is difficult to obtain, and it will be only after a very large number of cases have been treated that a proper judgment can be formed. On the whole the results obtained by the use of Washbourne's serum have been encouraging. Both from clinical and bacteriological evidence then it seems probable that lobar-pneumonia should be regarded as a general infection, and should be placed in the class of acute specific fevers.

